# Draft Genome Sequences of *Campylobacterales* (*Epsilonproteobacteria*) Obtained from Methanogenic Oil Sands Tailings Pond Metagenomes

**DOI:** 10.1128/genomeA.01034-14

**Published:** 2014-10-16

**Authors:** BoonFei Tan, Julia Foght

**Affiliations:** Department of Biological Sciences, University of Alberta, Edmonton, Alberta, Canada

## Abstract

Draft genome sequences of two *Campylobacterales* (*Sulfurospirillum* sp. strain SCADC and *Sulfuricurvum* sp. strain MLSB [Mildred Lake Settling Basin]) were obtained by taxonomic binning of metagenomes originating from an oil sands tailings pond. Both genomes contain *soxABXYZ* genes involved in sulfur oxidation, highlighting their potential roles in sulfur cycling in oil sands tailings ponds.

## GENOME ANNOUNCEMENT

Environmental sulfur-oxidizing *Epsilonproteobacteria*—specifically, members of the order *Campylobacterales*—are reported to dominate the deep subsurface of a severely biodegraded oil sands reservoir (1). However, their presence and importance have not been documented in the tailings ponds that store wastes from mining the oil sands’ ores (2–4).

The metagenome of MLSB (Mildred Lake Settling Basin), an oil sands tailings pond located in northern Alberta, Canada, was obtained by isolating total DNA for high-throughput sequencing using Illumina-HiSeq. Reads were subjected to quality control and sequence assembly using CLC Genomics Workbench version 7 (CLC-Bio, USA). Contigs assembled from the MLSB metagenome and from the metagenome of SCADC (a methanogenic alkane-degrading enrichment culture derived from MLSB [5]) were binned using sequence homology and composition-based methods, including sequence coverage, GC content, and contig length, followed by tetranucleotide frequency and analysis of principal components. Two *Campylobacterales*-associated genomic bins were recovered and subjected to sequence decontamination as previously described (6). Open-reading frames (ORFs) were predicted using Prodigal (7), followed by BLASTx searches against the NCBI NR database and taxon assignment using MEGAN version.5.0 (8). All contigs in both bins had ≥50% of their ORFs affiliated with *Campylobacterales*.

BLASTn comparison of 16S rRNA genes in the SCADC *Campylobacterales* bin indicated that the draft genome is closely (99%) related to *Sulfurospirillum multivorans* DSM 12446 (CP007201). The genome, hereby named *Sulfurospirillum* sp. strain SCADC, is ~2.7 Mbp contained in 38 scaffolds (680–290,000 bp) with average 41% GC content. All 107 single copy genes (ScpG) (9) had best BLAST hits to *Sulfurospirillum*; comparison to *Sulfurospirillum deleyianum* DSM 6946 (2.3 Mbp; CP001816.1; 108 ScpG) and *S. multivorans* DSM 12446 (3.2 Mbp; CP007201.1; 109 ScpG) indicated recovery of a nearly complete genome, even though the reference genomes have a broad size range (2.3 to 3.2 Mbp). Annotation using RAST (10) predicted 2,718 coding DNA sequences (CDSs) in *Sulfurospirillum* SCADC, with 1,695 of 2,718 ORFs sharing >60% BLASTp similarity with those in *S. deleyianum* DSM 6946, whereas 556 ORFs were present only in *Sulfurospirillum* SCADC.

Genome binning of *Campylobacterales*-affiliated contigs from MLSB did not recover any 16S rRNA genes. However, the draft genome contains 107 of 110 ScpG in *Sulfuricurvum kujiense* DSM 16994 (NC_014762.1), all of which had best BLAST hits to *Sulfuricurvum* spp., indicating recovery of a nearly complete genome. The draft genome (herein named *Sulfuricurvum* sp. MLSB) is ~2.1 Mbp contained in 148 scaffolds (1000–104,000 bp) with average 49% GC content. Of 2,119 predicted CDSs, 1,549 of 2,119 have BLASTp similarity >60% to ORFs in *S. kujiense* DSM 16994, whereas 277 CDSs were present only in *Sulfuricurvum* MLSB.

*Sulfurospirillum* SCADC and *Sulfuricurvum* MLSB both carry *soxABXYZ* genes for thiosulfate oxidation, plus genes for the Wood-Ljungdahl CO_2_ fixation pathway (11). Physiological examination of reference strains shows metabolic potential for using sulfur compounds, nitrate, or H_2_ as electron acceptors (12, 13), which are relevant to the biogeochemistry of oil sands tailings ponds (2, 4). Comparative genomics should provide insight into their importance in nutrient cycling and oxidation of reduced sulfur compounds in oil sands tailings ponds.

### Nucleotide sequence accession numbers.

The whole-genome shotgun projects of *Sulfurospirillum* sp. SCADC and *Sulfuricurvum* sp. MLSB have been deposited at DDBJ/EMBL/GenBank under the accession numbers JQGK00000000 and JQGL00000000, respectively. The versions described in this paper are versions JQGK01000000 and JQGL01000000.
